# Supplementary material for: Integrative Genetic and Single‐Cell Analysis Reveals Macrophages as Key Mediators Linking Aging and Osteoporosis
Source: Aging Cell. 2026 Nov 6;25(11):e70812. doi: 10.1111/acel.70812 (PMC13743511; doi:10.1111/acel.70812)
Supplement: Supplementary file 1 [file ACEL-25-e70812-s001.docx]

Supplementary Figure 1. Sensitivity analyses to evaluate the robustness of the MR results. Q_pval is the pvalue of Cochran’s Q test. Q_pval < 0.05 suggest heterogeneity. MR-Egger regression and MR-PRESSO are used to identify horizontal pleiotropy. If P_pleiotrogy (Egger) and P_pleiotrogy (Presso) > 0.05, there is no evidence of horizontal pleiotropy.

Supplementary Figure 2. UMAPs of bone remodeling cell types. (A) UMAP of cell types related to immune cells, MSCs, osteoblasts differentiated from MSCs and others. (B) UMAP of cell types related to osteoclastogenesis.

Supplementary Figure 3. Benchmarking of integration using different methods on single-cell sequencing data. (A) Quantitative evaluation of 5 integration methods (Combat, Harmony, scANVI, scVI and BBKNN) across three categories: Biological conservation, Batch correction and Aggregate scores. (A) UMAP of integrated embeddings generated by each method. For each panel, left plot shows cell clustering colored by batch origin, while right plot shows shared cell types to assess biological structure preservation.

Supplementary Figure 4. Integrative analysis of human bone marrow niche single-cell transcriptomics and GWAS summary statistics as a human validation. (A) UMAP visualization of annotated cell types of human bone marrow niche. (B) scGWAS analysis revealing cell type-specific enrichment for bone aging-related traits. Scatter plots show the enrichment of GWAS summary statistics for mvAge (left panel) and eBMD (right panel) across different cell types, with red dots indicating significant genetic enrichment.

Supplementary Figure 5. Marrow macrophage percentages at different ages. (A) Macrophage percentages across different ages, calculated with the annotation information provided in the original study. (B) Macrophage percentages in heterochronic parabiosis mice at different ages.

Supplementary Figure 6. Characterization of macrophage subpopulations in mouse bone marrow niche. (A) UMAP visualization of macrophage heterogeneity, revealing five distinct clusters: IFN-simulated Macrophages, Inflammatory Macrophages, Proliferating Macrophages, MHC-II Macrophages and Lipid-associated Macrophages; (B) Dot plot illustrating the expression profiles of canonical marker genes across the identified macrophage subpopulations.

Supplementary Figure 7. Shared genetic tools identify pleiotropic loci between mvAge and eBMD.

Supplementary Figure 8. Characterization of macrophage subpopulations and their dynamics across age. (A) Dot plot illustrating the expression profiles of canonical marker genes of M1/M2 polarization across the identified macrophage subpopulations. (B) Line plots visualizing the positive correlation between *HNRNPUL1* or *TGFB1* expression levels and the scores for "Proliferating" and "MHC_II" macrophage subclusters. (C) Ridge plots and scatter plots depicting the expression trajectories of specific marker genes as a function of Age. The left panel highlights markers for MHC-II macrophages (*Ccr2*, *Cd74*, *H2-Ab1*), while the right panel shows markers for Proliferating macrophages (*Ccna2*, *Cdk1*, *Mki67*, *Stmn1*).

**Legends for supplementary tables**

Supplementary Table 1. Description of phenotypic traits and their corresponding Field IDs. Fracture data were obtained from three sources: self-reported questionnaires (*), ICD-10 hospital inpatient records (†), and ICD-9 hospital inpatient records (‡). Heel bone mineral density (heel BMD) was measured via quantitative ultrasound. Abbreviations: ICD, International Classification of Diseases.

Supplementary Table 2. Overview of GWAS and eQTL datasets used in the analysis. All GWAS summary statistics are predominantly based on individuals of European descent unless otherwise specified. Abbreviations: eBMD, estimated bone mineral density; eQTL, expression quantitative trait locus; mvAge, multivariate age.

Supplementary Table 3. Summary of single-cell transcriptomics datasets utilized in this study. Full citations for the original studies are provided.

Supplementary Table 4. Parameters and filtering criteria for two-sample Mendelian Randomization analyses. The table summarized the configuration settings used for each exposure-outcome pair.

Supplementary Table 5. Mean gene expression per cell type for integrated expression atlas. Expression levels were normalized using scVI and scANVI.

Supplementary Table 6. Significant modules identified using scGWAS. Modules with *p* < 0.05, *p_GWAS* < 0.05 and *p_scRNA* < 0.05 were significant. Macrophages were the only cell type harboring modules significantly associated with both traits.

Supplementary Table 7. Significant gene modules identified by scGWAS in macrophage subpopulations. The table lists gene modules significantly associated with aging (mvAge) and bone mineral density (eBMD)

Supplementary Table 8. Gene list of the Gainsboro module. Gainsboro module is enriched for genes whose expression is strongly correlated with macrophages ratios across samples.

Supplementary Table 9. Gene Ontology (GO) enrichment analysis of the Gainsboro gene list obtained by pyWGCNA. Terms are ranked by their Combined Score.

Supplementary Table 10. Gene loci exhibiting a conjunctional false discovery rate (conjFDR) < 0.05 for both mvAge and eBMD, indicating shared genetic associations with these two risk factors.

Supplementary Table 11. Genomic regions showing significant genetic correlation (rho) between mvAge and eBMD. Regions were considered correlated if *p* < 0.05, reflecting shared regulatory pleiotropy.

Supplementary Table 12. Characterization of HNRNPUL1 binding sites on the TGFBR2 transcript. The table details specific RNA-protein interaction events identified via enhanced Cross-Linking and Immunoprecipitation (eCLIP) in HepG2 cells. Data were sourced from the ENCODE project.
